# Application of a New Multiplexed Array for Rapid, Sensitive, Simultaneous and Quantitative Assessment of Spliced and Unspliced XBP1

**DOI:** 10.1186/s12575-019-0111-3

**Published:** 2019-11-15

**Authors:** Stuart Creedican, Aaron Talty, Stephen P. Fitzgerald, Afshin Samali, Ciarán Richardson, Adrienne M. Gorman, Kenneth Martin

**Affiliations:** 1 Randox Teoranta, Meenmore, Dungloe, Co. Donegal Ireland; 2 Apoptosis Research Centre, National University of Ireland Galway, University Road, Galway, H91 TK33 Ireland; 3 Randox Laboratories Ltd., 55 Diamond Road, Crumlin, Co. Antrim Northern Ireland BT29 4QY

**Keywords:** UPR, XBP1, Protein biochip, Quantitative XBP1 assessment

## Abstract

**Background:**

IRE1α-mediated unconventional splicing of XBP1 is emerging as a biomarker in several disease states and is indicative of activation of the unfolded protein response sensor IRE1. Splicing of *XBP1* mRNA results in the translation of two distinct XBP1 protein isoforms (XBP1s and XBP1u) which, due to post-translational regulation, do not correlate with mRNA levels. As both XBP1 isoforms are implicated in pathogenic or disease progression mechanisms there is a need for a reliable, clinically applicable method to detect them.

**Methods:**

A multiplexed isoform-specific XBP1 array utilising Biochip array technology (BAT™) was assessed for specificity and suitability when using cell protein lysates. The array was applied to RIPA protein lysates from several relevant pre-clinical models with an aim to quantify XBP1 isoforms in comparison with RT-PCR or immunoblot reference methods.

**Results:**

A novel reliable, specific and sensitive XBP1 biochip was successfully utilised in pre-clinical research. Application of this biochip to detect XBP1 splicing at the protein level in relevant breast cancer models, under basal conditions as well as pharmacological inhibition and paclitaxel induction, confirmed the findings of previous studies. The biochip was also applied to non-adherent cells and used to quantify changes in the XBP1 isoforms upon activation of the NLRP3 inflammasome.

**Conclusions:**

The XBP1 biochip enables isoform specific quantification of protein level changes upon activation and inhibition of IRE1α RNase activity, using a routine clinical methodology. As such it provides a research tool and potential clinical tool with a quantified, simultaneous, rapid output that is not available from any other published method.

## Introduction

The unfolded protein response (UPR) is a set of eukaryotic pathways activated by endoplasmic reticulum (ER) stress, which is defined by an accumulation of unfolded or misfolded proteins in the ER [1]. Canonically, detection of protein misfolding is dependent upon three ER-transmembrane receptors: inositol requiring enzyme 1α (IRE1α), protein kinase RNA-activated (PKR)-like ER kinase (PERK) and activating transcription factor 6 (ATF6) [2]. The most studied and characterised of these ER stress sensors is IRE1. Activation of the ubiquitous IRE1α isoform is dependent upon its autophosphorylation and subsequent oligomerisation which results in activation of its cytosolic RNase domain [3]. Signal transduction is possible through both kinase and RNase activity [4]. The most widely studied result of IRE1α activation is the RNase-mediated unconventional splicing of *XBP1* pre-mRNA, resulting in the excision of 26 nucleotides and a frameshift in its open reading frame [5–7].

Translation of the conventionally spliced mRNA, *XBP1u*, results in a 29 kDa 232 amino acid protein, XBP1u, which has few known functions and is rapidly degraded in vitro [8, 9]. The most characterised action of XBP1u is as a negative regulator of XBP1s, though it has also been linked to regulation of the p53/p21 tumour suppression pathway [9, 10]. In contrast, translation of *XBP1s* mRNA (the result of unconventional IRE1α mediated *XBP1* splicing) produces a potent transcription factor of 261 amino acids and ~ 55 kDa (Additional file 1: Figure S1A). XBP1s, along with other UPR regulated transcription factors, initiates a transcriptional programme aimed at reducing protein load through increased expression of the ER’s protein folding or protein degradation machinery [11]. Increased splicing of *XBP1* has been associated with disease progression, therapy resistance and as a druggable target in a range of diseases [1].

The UPR is activated as a key pro-survival mechanism in many solid tumours in response to hypoxic and nutrient deprived conditions [1]. Constitutive activation of IRE1α is proposed to confer a selective advantage onto cancer cells over neighbouring healthy and non-UPR activated cancer cells, with recent studies demonstrating upregulated *XBP1* splicing in breast, pancreatic and ovarian cancer [12–14]. XBP1s upregulation in immune cells also contributes to immune evasion within the tumour microenvironment [15, 16]. Several conventional therapies routinely used in cancer treatment induce IRE1α RNase activity, either providing pro-survival resistance or enhancing apoptotic effects [12, 17]. Small molecule mediated targeting of IRE1α RNase activity is being investigated as an adjuvant therapy in several cancers [12, 18–20]. XBP1s has been implicated in the pathology in neurodegenerative disease models including Alzheimer’s, Parkinson’s and Huntington’s diseases [21]. The consequences of IRE1α activation are highly context dependent, with links to various molecular pathways including autophagy, apoptosis and prion resistance [22–24].

As therapies targeting the UPR enter clinical trials, and evidence for the use of XBP1s as a pathologically relevant biomarker grows, effective means of monitoring XBP1 splicing and expression of the XBP1 isoforms has become a clinical need. None of the methods currently employed for XBP1s or XBP1u detection are suitable for routine use in a clinical laboratory [25]. RT-PCR and RT-qPCR are often used to assess *XBP1* splicing, using primers flanking the spliced intron sequences where variant specificity is required [12]. Whilst more specialised laboratories can utilise RT-qPCR to obtain a quantitative measurement of XBP1s/XBP1u ratios, this method would have far less reliable results in a routine clinical setting. Factors such as extended sample preparation, potential for contamination and requirements for standardisation and normalisation of results make RT-qPCR unsuitable for medium-high throughput in non-sterile clinical laboratories [25]. At the protein level, standard assessment of XBP1s (and less commonly XBP1u) is performed by immunoblotting. Medium to high throughput is not practical with this time-consuming and technically laborious method. Western blots are also largely unsuitable for quantification or inter-blot comparison with variation resulting from detection mechanisms, reagents and analysis methods [26–28].

Biochip Array Technology (BAT^TM^) is commonly used in both clinical and research settings to simultaneously, quantitatively determine protein levels in serum and other biological matrices. The multiplexed sandwich immunoassay system provides a platform to assess multiple protein levels in a single sample [29, 30]. An assay system was proposed using the unique principles of sandwich-BAT to simultaneously capture the two XBP1 isoforms utilising their different C-termini, and detecting the captured protein using a Horse Radish Peroxidase (HRP) conjugated pan-detector targeted to their common N-terminus (Additional file 1: Figure S1B). Here we demonstrate the application of a biochip to simultaneous detection of the two XBP1 isoforms in models of breast cancer, non-adherent cells and inflammation.

## Materials and Methods

### Reagents

XBP1 Biochip assay kit was obtained from Randox Laboratories (XBP1 Array (EV4357), Randox Laboratories Ltd., Crumlin, UK). Thapsigargin (Tg) (Sigma-Aldrich, St. Louis, USA), Tunicamycin (Tm) (Sigma-Aldrich, St. Louis, USA), Brefeldin A (BFA) (Sigma-Aldrich, St. Louis, USA), Dithiothreitol (DTT) (Sigma-Aldrich, St. Louis, USA), 4μ8C (Sigma-Aldrich, St. Louis, USA), MKC-8866 (Probechem, St Pete Beach, USA), Paclitaxel (Taxol) (Sigma-Aldrich, St. Louis, USA), Lipopolysaccharide (LPS) (Sigma-Aldrich, St. Louis, USA) and Nigericin (Nig) (InvivoGen, San Diego, USA) were diluted in DMSO (Sigma-Aldrich, St. Louis, USA).

### Biochip-Based Determination of XBP1s and XBP1u

XBP1s and XBP1u levels and raw signal were quantified using the Evidence Investigator analyser (EV3602, Randox Laboratories Ltd., UK). Signals from defined discrete test regions (DTRs) were detected using digital imaging technology as previously described [31]. The biochips were provided in wells within a carrier in a 3 × 3 format (9 reaction wells per carrier). A carrier handling tray supplied with the system accommodated 6 carriers. The total assay protocol was performed according to manufacturer’s instructions.

Briefly, RIPA lysed samples were diluted to 100 μL in RIPA buffer followed by dilution to 200 μL in XBP1 assay buffer and mixed well with gentle pipetting. Two hundred microliter of XBP1 assay diluent was then applied to the surface of the biochip followed by 200 μL diluted sample or 100 μL calibrator/control per well. Following a 60 min incubation at 37 °C, 370 RPM in a thermoshaker (Randox Laboratories Ltd., Crumlin, UK) liquid contents were removed with a sharp flick and washed in a 2X quick, 4X 2 min wash steps. Three hundred microliter of HRP-conjugated pan-XBP1 detector was applied to each well and another 60 min incubation at 37 °C, 370 RPM in a thermoshaker was performed. Following another wash as above 250 μL of a 1:1 ratio of EV841 luminol and peroxide was applied to the surface of each biochip in a carrier and incubated away from direct light for 2 min. Carriers are then submitted to the Evidence Investigator, light emitted from each DTR is detected by the CCD camera and signal quantified by the instrument software.

Final values in pg/mg were obtained by dividing reported value (in pg) by total protein loaded per well, as determined by bicinchoninic acid (BCA) assay. Calibration curves, inferred values and goodness of fit were independently confirmed using R package nplr (0.1–7, Frederic Commo, https://cran.r-project.org/web/packages/nplr/index.html).

### Cell Culture and Treatments

MCF10A (ATCC) cells were maintained in DMEM/F-12 (Sigma-Aldrich, St. Louis, USA) supplemented with 5% horse serum (Sigma-Aldrich, St. Louis, USA), 20 ng/mL epidermal growth factor (PeproTech, London, UK), 0.5 μg/mL hydrocortisone (Sigma-Aldrich, St. Louis, USA), 100 ng/mL cholera toxin (Sigma- Aldrich, St. Louis, USA), 10 μg/mL insulin (Sigma-Aldrich, St. Louis, USA), 50 U/mL penicillin, and 50 μg/mL streptomycin (St. Sigma-Aldrich, St. Louis, USA). MCF7 cells (ECACC) were cultured in DMEM high glucose (Sigma-Aldrich, St. Louis, USA) supplemented with 10% foetal bovine serum (FBS), 0.01 mg/mL insulin (Sigma-Aldrich, St. Louis, USA), and 2 mM L-glutamine. MDA-MB-231 cells were cultured in DMEM high glucose (Sigma-Aldrich, St. Louis, USA) supplemented with 10% FBS, 50 U/mL penicillin, 50 μg/mL streptomycin, and 2 mM L-glutamine. U937 and THP-1 (ATCC, Manassas, USA) cells were cultured in RPMI-1640 medium (Sigma-Aldrich, St. Louis, USA) supplemented with 10% FBS (Sigma-Aldrich, St. Louis, USA), 50 U/mL penicillin, 50 μg/ml streptomycin (Sigma-Aldrich, St. Louis, USA), and 2 mM L-glutamine (Sigma-Aldrich, St. Louis, USA). All cells were cultured at 37 °C, 5% CO_2_ in a humidified incubator and adherent cells seeded at an appropriate density 24 h prior to treatment. U937 cells were seeded at 5X10^5^ cells/mL and treated immediately while THP-1 cells were seeded at 1X10^6^ cells/mL and treated after 2 h.

### Sample Preparation

Cells were washed once in ice cold phosphate-buffered saline (PBS) and then lysed in either RIPA buffer (Sigma-Aldrich, St. Louis, USA) with RocheSTOP® protease inhibitors (Roche, Basel, Switzerland) for protein analysis or directly lysed in TriReagent (Sigma-Aldrich, St. Louis, USA) as per manufacturer’s instructions for RNA analysis.

### Immunoblotting

Protein lysates were mixed with 5X Laemmli Buffer (0.3125 M Tris HCl (pH 6.8), 10% SDS, 50% glycerol, 25% 2-mercaptoethanol, 0.02% bromophenol blue) in a 1:4 ratio and boiled at 95 °C for 5 min. Samples were separated on an SDS polyacrylamide gel, transferred onto nitrocellulose membrane (GE Healthcare Life Sciences, Little Chalfont, UK) and blocked with 5% milk in Wash Buffer (Randox Laboratories Ltd., Crumlin, UK). Membranes were probed with commercial antibodies to spliced and unspliced XBP1 isoforms and actin (Sigma-Aldrich, St. Louis, USA, 1:5000). Anti-rabbit (Jackson ImmunoResearch, Cambridge, UK) and anti-mouse (Jackson ImmunoResearch, Cambridge, UK; Sigma-Aldrich, St. Louis, USA) HRP-conjugated secondary antibodies were incubated for 45–60 min and the signal was visualized using western blotting luminol reagent (Thermofisher, Waltham, USA; Perkin-Elmer, Waltham, USA).

### RT-PCR

500–5000 ng of purified RNA was reverse transcribed using Superscript II (Thermofisher, Waltham, USA). PCR was performed using GoTaq Green (MyBio Ltd., Kilkenny, Ireland) master mix and the following primers: FW XBP1 *5′-GGA ACA GCA AGT GGT AGA-3′*, RV XBP1 *5′-CTG GAG GGG TGA CAA CTG-3′*, FW GAPDH 5′-ACC ACA GTC CAT GCC ATC-3′ and RV GAPDH *5′-TCC ACC ACC CTG TTG CTG-3′.* Products were visualised on 3–4% Agarose in Tris Base, Acetic Acid, EDTA (TAE) buffer gels and stained with Midori green (ANACHEM, Leicester, UK).

### Statistical Analysis

Statistical analysis was carried out using two-tailed t test with Welch’s correction or one-way ANOVA where appropriate. *P* < 0.05 was considered statistically significant. Final analysis and calculations were performed in R version 3.5.1 “Feather Spray”.

## Results

### BAT multiplexed XBP1s and XBP1u assays are specific to their respective antigen and produce antigen dependent signal

**Fig. 1:**
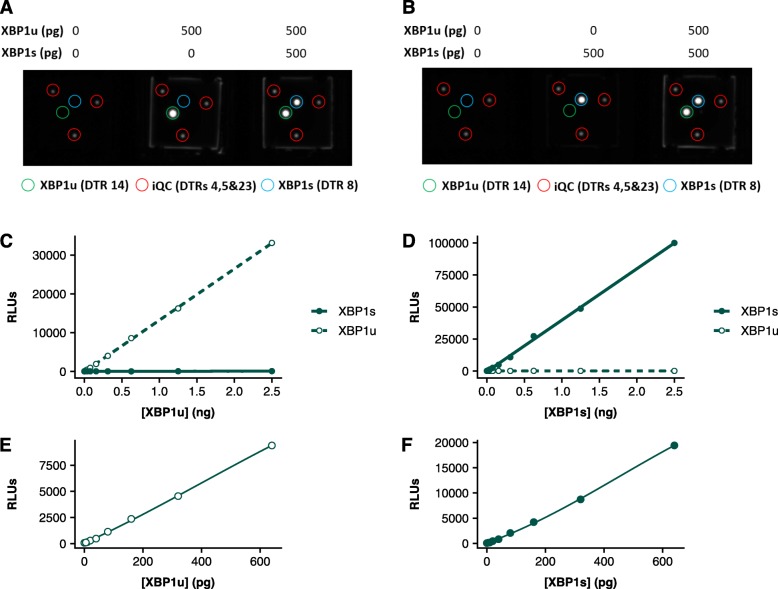
*XBP1 biochip specificity and signal-antigen relationship upon XBP1s and XBP1u spiking and induction*. 500 pg XBP1s and/or XBP1u were spiked alone or in combination into calibrator base material before application to XBP1 biochip. DTRs are indicated, including those used for internal quality control (iQC) (**a**, **b**). XBP1s and XBP1u were individually spiked into calibrator base material at 2.5 ng per sample and serially diluted before application to XBP1 biochip (**c**, **d**). XBP1 biochip multiplexed calibration curves from 0 to 650 pg per sample (**e**, **f**)

To assess the XBP1 biochip’s suitability and performance as a sandwich immunoassay in a multiplexed format the specificity and sensitivity of each assay was assessed. Sandwich BAT assays utilise a solid state immobilised multiplexed ELISA based system as described previously [30]. BAT multiplexing of the two XBP1 isoforms requires the spotting of the two identified isoform specific capture antibodies into distinct DTRs on the ceramic biochip surface. Of the 25 DTRs present on a conventional biochip array three are used for internal quality control by the analysis software (DTRs 4, 5 and 23). DTRs 8 and 14 were chosen for XBP1u (Fig. 1a) and XBP1s analysis (Fig. 1b), respectively.

Clear signal with minimal background and cross-reactivity was observed upon spiking recombinant XBP1u (Fig. 1a) or XBP1s (Fig. 1b) into XBP1 assay calibrator base and assessing signal at the corresponding and other analyte’s DTR. This DTR specificity and acceptable background signal (≤100 relative light units (RLU)) was observed even at 2.5 ng for both XBP1u (Fig. 1c) and XBP1s (Fig. 1d, Additional file 2: Table S1). When spiked with 6.5 ng of XBP1u or XBP1s, cross reactivity at the other DTR was determined to be 0.61 and 0.30%, respectively. It was possible to obtain a reliable, antigen dependent signal with consistent and reproducible RLU observed at a 0–650 pg calibration range (Fig. 1e, f). The analytical sensitivity of each analyte was determined across 27 replicates and found to be 4.13 pg and 3.40 pg of XBP1u and XBP1u, respectively, while the functional sensitivity of each assay was 5.00 and 9.70 pg, respectively. Multiplexed intra-assay precision was determined at two levels (~ 15–20 pg and ~ 55–100 pg) for each analyte and was below 10% for XBP1s and 15% for XBP1u across 27 replicates. These results demonstrate that the XBP1 biochip can reliably quantify the XBP1 isoforms with high specificity.

### XBP1 Biochip Detects XBP1 Splicing Upon Pharmacologically-Induced ER Stress

**Fig. 2:**
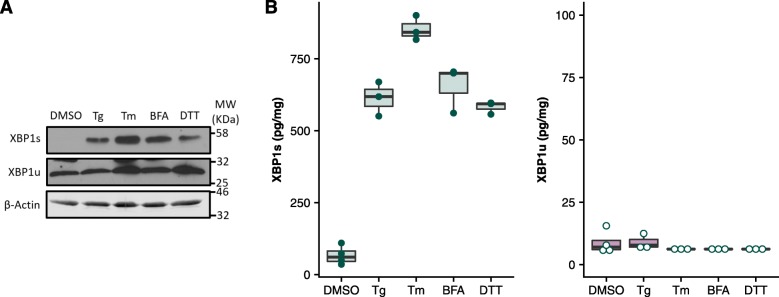
*Quantified XBP1s upregulation upon pharmacologically induced ER stress*. MDA-MB-231 cells were treated with UPR inducers Tg (0.5 mM, 48 h), Tm (1 μg/ml, 16 h), BFA (0.5 μg/ml, 16 h) and DTT (4 mM, 2 h) before being assessed by immunoblot (**a**) and XBP1 biochip (**b**)

To confirm utility of the XBP1 biochip in cell lysates, a common model used to investigate ER stress mechanisms was utilised. Pharmacological induction of XBP1 splicing in in vitro and in vivo model systems is normally performed using a number of drugs with different mechanisms of action, all of which lead to an accumulation of misfolded proteins in the ER [32]. When MDA-MB-231 triple negative breast cancer (TNBC) cells were treated with Thapsigargin (Tg), Tunicamycin (Tm), Brefeldin A (BFA) or Dithiothreitol (DTT) an upregulation in XBP1s translation was observed (Fig. 2a). The XBP1 biochip was able to quantify the changes in XBP1 isoform expression, detecting the resultant XBP1 splicing at the protein level in RIPA lysates (Fig. 2b). A 10–30-fold increase in XBP1s/XBP1u ratio was detected by the XBP1 biochip when cells were treated with these commonly used treatments (Additional file 3: Figure S2) [12, 33, 34]. This confirms the XBP1 biochip is also suitable for detection and analysis of pharmacologically induced XBP1 splicing using treatments commonly utilised in investigating the effects of UPR activation.

### XBP1 Assessment Using BAT in Relevant Pre-Clinical Breast Cancer Cell Models

In order to test the XBP1 biochip in a physiologically relevant model, basal XBP1 levels were assessed in several breast cancer cell lines of different subtypes. Recent studies have shown constitutive activation of IRE1α and resultant *XBP1* splicing in basal-like (when stratified molecularly) and triple negative (when stratified by receptor expression) breast cancers. Other breast cancer subtypes can also display basal IRE1α activity, but to a lesser extent [12].

**Fig. 3:**
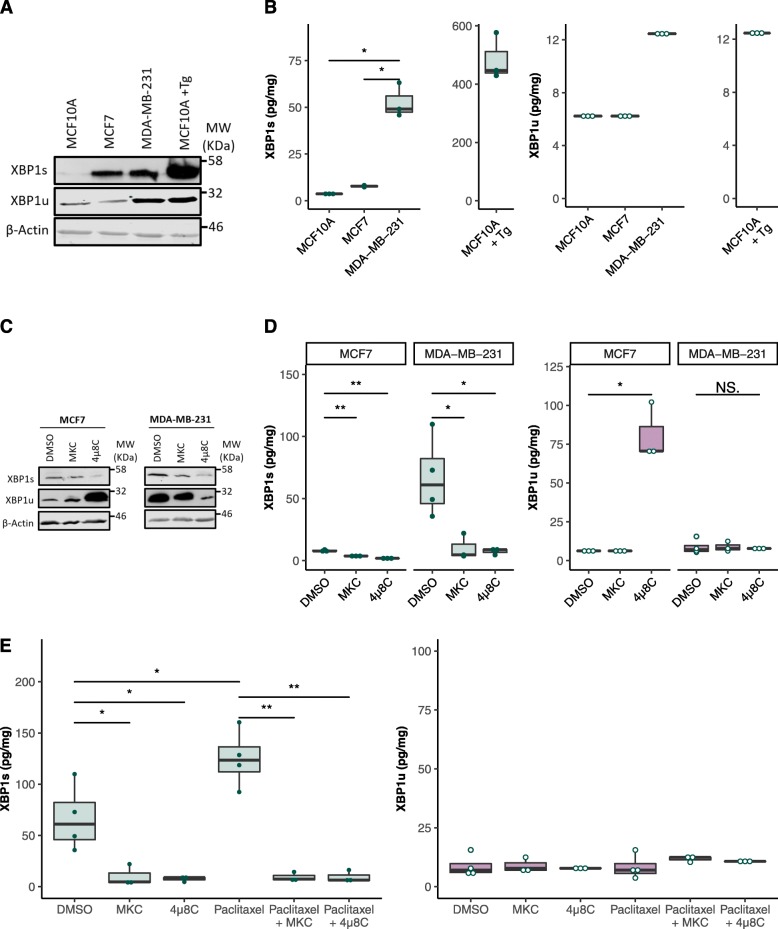
*Basal and treatment dependent variation in XBP1 levels were quantified using the XBP1 biochip*. XBP1s and XBP1u levels in MCF7, SKBR3 and MDA-MB-231 cell lines were assessed by immunoblot (**a**) and XBP1 biochip (**b**). MCF10A (± 0.5 mM Tg) were included as negative and positive controls. * *p < 0.05.* MCF7 and MDA-MB-231 cells were treated with vehicle (DMSO) or IRE1α RNase inhibitors 4μ8C (32 μM) or MKC-8866 (20 μM) for 48 h and assessed by immunoblot (**c**) and XBP1 biochip (**d**). XBP1 biochip assessment of MDA-MB-231 cells following 48 h treatment with XBP1 splicing inducing chemotherapeutic Paclitaxel (10 nM) and IRE1α RNase inhibition (**e**)

Immunoblots confirmed previous results in XBP1 isoforms when comparing non-tumourigenic, Oestrogen receptor positive (ER+) and triple negative breast cancer cell lines (MCF10A, MCF7 and MDA-MB-231, respectively) (Fig. 3a). Using the XBP1 biochip we were able to observe and quantify these differences in XBP1 splicing at the protein level. TNBC cell line MDA-MB-231 displayed significantly higher basal XBP1 splicing than the non-tumourgenic or ER+ cell lines (*p* = 0.014 and 0.012 respectively). MCF7 cells also showed low level IRE1α activity, with a mean XBP1s expression of 7.78 pg/mg (S.E. = 0.278 pg/mg). Reported XBP1u levels were consistently close to or at the functional sensitivity of the assay of 9.70 pg, indicating low basal expression levels (Fig. 3b).

To test if the XBP1 biochip has suitable sensitivity for research applications, IRE1α RNase activity was pharmacologically inhibited. Inhibition of basal IRE1α RNase activity has previously been shown to reduce tumour progression and size in vitro and in vivo in murine models [12]. Immunoblot was used to confirm a reduction in XBP1s both in MDA-MB-231 and MCF7 cells (which exhibit high and low basal levels of XBP1s, respectively) when using IRE1α RNase inhibiting compounds 4μ8C and MKC-8866 (Fig. 3c). When quantified by the XBP1 biochip, 4μ8C appeared to be more potent than MKC-8866, but use of both compounds resulted in significant reduced XBP1s in MCF7 (*p* = 0.004 and *p* = 0.008) and MDA-MB-231 cells (*p* = 0.035 and *p* = 0.033). XBP1u protein levels remained largely unchanged in the MDA-MB-231 cells during IRE1α inhibition (*p* = 0.71). However, MCF7 cells showed a significant upregulation of XBP1u protein upon 4μ8C treatment (*p* = 0.019) with expression levels averaging 81.1 pg/mg (Fig. 3d).

To assess the biochip’s applicability in pre-clinical models a currently clinically approved modulator of IRE1α activity was used. Paclitaxel, a commonly used chemotherapeutic in TNBC treatment, has also been shown to induce *XBP1* mRNA splicing [12]. Here we demonstrated that this increase in XBP1s was observed at the protein level, and that drug-induced changes in XBP1s were quantifiable with the XBP1 biochip. Paclitaxel treatment of MDA-MB-231 cells resulted in a modest but significant increase in XBP1s expression (66.9 ± 16.2 pg/mg to 125.0 ± 14.1 pg/mg, *p* = 0.036). This increase was completely ablated by pharmacological inhibition of IRE1α RNase activity (*p* < 0.01 upon MKC-8866 or 4μ8C treatment). XBP1u levels showed no significant change with Paclitaxel combined with MKC-8866 or 4μ8C treatment (Fig. 3e).

These data indicate the utility of the XBP1 biochip in relevant pre-clinical models, showing its ability to detect basal and treatment induced levels of the XBP1 isoforms. Quantification of treatment-induced changes resulted in significant differences in XBP1s levels of cells treated with IRE1α inhibitors MKC-8866 and 4μ8C, and the clinically relevant ER stress inducer, Paclitaxel.

### The XBP1 Biochip Shows Increased Ease of Use and Sensitivity in Non-adherent Model Systems

**Fig. 4:**
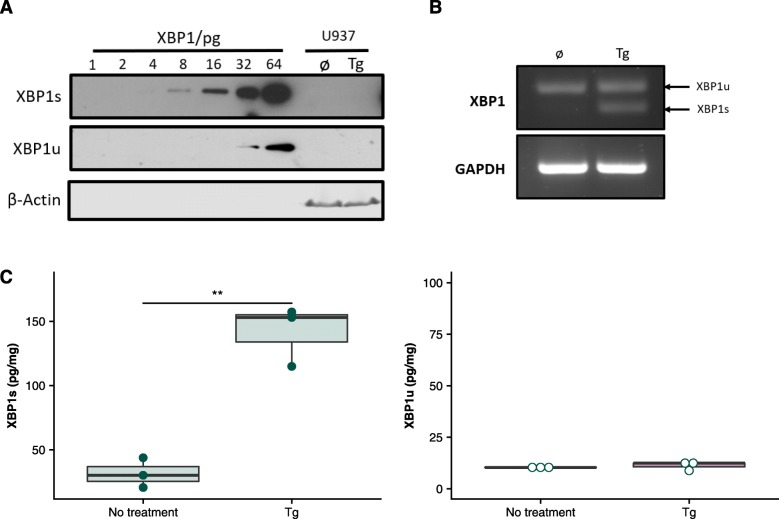
*BAT*^*TM*^ *technology detection of XBP1s and XBP1u levels in U937 lysates*. 2.5X10^6^ untreated and Tg (0.25 mM, 48 h) treated U937 cells were lysed in RIPA and immunoblotted for XBP1s and XBP1u alongside a serial dilution of His-tagged recombinant protein. β-Actin acts as a loading control (**a**). RNA was extracted from the same cells and analysed by RT-PCR for XBP1 spicing. GAPDH serves as a control (**b**). RIPA lysed U937 cells were treated as in **(a)** and assessed by XBP1 biochip. ** *p < 0.01* (**c**)

To determine if the XBP1 biochip had advantages over immunoblotting (the current standard method of protein level detection) a non-adherent cell model was assessed. Detecting the XBP1 isoforms by immunoblotting can be difficult, with many researchers preferring to detect *XBP1* splicing at the mRNA level [35]. Assessment of proteins of low abundance by immunoblotting can be particularly difficult in non-adherent cells [36]. This difficulty is typified by U937 cells, where, even after achieving exceptionally high sensitivity (low picogram levels), it was not possible to detect either XBP1 isoform in unstimulated or Tg stimulated samples by immunoblot, even with high protein input and prolonged exposure of the X-ray film (Fig. 4a). Detection difficulty was not due to lack of *XBP1* expression or IRE1α RNase activity, as both presence and splicing of the transcript were confirmed using RT-PCR (Fig. 4b). In contrast, the XBP1 biochip was able to detect significant changes in XBP1s protein expression in the same samples (*p* = 0.0056, Fig. 4c).

This demonstrates that the XBP1 biochip can be used to assess protein levels of both XBP1 isoforms even in model systems where XBP1 immunoblots can be difficult. The immunoblots shown here took approximately 72 h whilst total assessment using the XBP1 biochip took ~ 3 h.

### NLRP3 Inflammasome Activation Associated XBP1 Splicing Is Detectable at the Protein Level with BAT^TM^

To confirm the applicability of the XBP1 biochip in a relevant non-adherent model system pro-monocytic THP-1 cells were assessed upon inflammatory release. Talty et al. recently demonstrated that XBP1s splicing occurs upon activation the NLRP3 inflammasome and that inhibition of IRE1α RNase activity could ablate NLRP3 mediated IL-1β release [37].

**Fig. 5:**
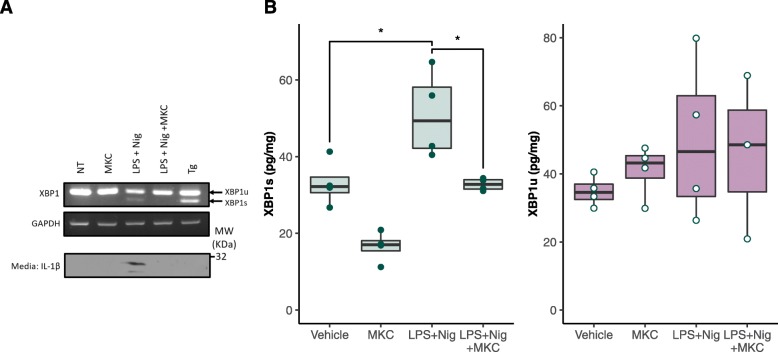
*Multiplexed XBP1 assessment following inflammasome activation***.** THP-1 cells were treated with LPS (1 μg/ml, 4 h) followed by nigericin (10 μM, 45 min), resulting in NLRP3 inflammasome activation and an increase in XBP1 splicing. Immunoblot of IL-1β in the conditioned media confirmed its release and inflammasome activation (**a**). XBP1 transcript and protein levels were assessed by RT-PCR **(a)** and biochip (**b**) respectively. * *p < 0.05*

Here we demonstrate that this XBP1s splicing is detectable after only 4 h of LPS priming and 45 min of Nigericin secondary signal treatment and we replicate the previous observation that MKC-8866 mediated inhibition of IRE1α RNase activity ablates IL-1β release (Fig. 5a). The XBP1 biochip was used to detect and quantify these effects on XBP1 splicing at the protein level, detecting significant changes in XBP1s expression upon inflammasome activation (*p* = 0.044) and MKC-8866 mediated inhibition (*p* = 0.048). XBP1u levels remained relatively high (mean = 46.2 pg/mg) but were largely unchanged by treatment (one-way ANOVA, *p* = 0.408) (Fig. 5b). Thus, the XBP1 biochip can detect XBP1 splicing following inflammatory stimulation and has identified a previously unreported high level of XBP1u protein in THP-1 cells.

## Discussion

Here we have shown a rapid, reliable and quantitative method of detecting the XBP1 isoforms simultaneously at the protein level in several relevant model systems. This is the first time the individual XBP1 isoforms have been quantitatively assessed using a BAT^TM^ platform which is already available in pre-clinical and clinical settings in laboratories worldwide. As the IRE1α mediated unconventional splicing of *XBP1* pre-mRNA, resulting in the differential expression of the XBP1 isoforms, emerges as a biomarker and a druggable target in various disease states, this method will potentially allow researchers and clinicians alike to quickly determine basal expression, prognostic utility in multiple diseases and predict drug efficacy [12, 19, 38].

Isoform specific differential recognition has been shown to be a powerful tool in the multiplexing of the XBP1 assays, allowing for simultaneous detection of XBP1s and XBP1u in a single sample, with no apparent cross-reactivity of the other analyte, even when tested at levels far exceeding those observed in any of the samples used during development. This demonstrates that BAT^TM^ is highly suited to multiplexed analysis of protein isoforms, even those of high similarity such as XBP1. The application of BAT^TM^ to detect protein isoforms has previously been demonstrated for simultaneous detection of ApoE isoforms in plasma, whilst the XBP1 biochip displays the utility of this technology in cell extracts [30]. Other enzyme-linked immunosorbent assay (ELISA) systems capable of XBP1 quantification are commercially available, but they fail to differentiate between the two isoforms and to our knowledge do not yet appear to have been used in any published works. In fact, use of these systems as an indirect measurement of XBP1s upregulation could lead to erroneous conclusions. This is demonstrated by MCF7 cells treatment with 4μ8C, where only an increase in total XBP1 would be observed and not a reduction in splicing and increased expression of XBP1u (Fig. 3d).

The XBP1 biochip has also demonstrated just how divergent protein levels of XBP1s and XBP1u are from their relative transcript levels. Whilst *XBP1s* transcript appears to correlate with protein levels of the isoform, XBP1u levels bear almost no resemblance to their transcript expression. In all but the most severe responses to ER stress *XBP1u* has remained the dominant transcript but at the protein level this pattern is reversed with much lower reported levels of XBP1u relative to XBP1s, particularly under ER stress [39]. Further exacerbating issues with this readout of IRE1α-mediated effects is the use of *XBP1s/XBP1u* mRNA ratios, that are commonly used as a readout of IRE1α activity in the literature [12]. At the protein level this ratio-based measurement of XBP1 isoform expression was suitable (though statistically less powerful) in the breast cancer models (Additional file 3: Figure S2 and Additional file 4: Figure S3) where XBP1u reported at low levels with little variation. A ratio measurement was also appropriate for Thapsigargin treated U937 cells (Additional file 5: Figure S4). However, this was not suitable for the THP1 model of inflammasome activation used (Additional file 6: Figure S5), where no significant changes in protein ratio were observed upon inflammasome activation despite the clear activation of IRE1α RNase activity and resultant XBP1s expression. For many researchers this method of using mRNA ratios (or, in some cases, percentage of *XBP1s* of total *XBP1*) has not been considered an issue (e.g., those only looking for a functional readout of IRE1α RNase activity). However, as the field develops and requires greater mechanistic explanations of downstream effects this will likely become an important issue for those requiring reliable, accurate measurement of the effector proteins of the IRE1-XBP1 pathway.

In spite of the low levels of both protein isoforms (relative to other conventional biomarkers and assays) it was still possible to quantify and detect significant changes in several relevant model systems. Quantification and simultaneous assessment also appeared to highlight the differential action of MKC-8866 and 4μ8C in MCF7 cells. The greater inhibition observed upon application of 4μ8C when compared with MKC-8866 is proposed to be due to the higher concentration used (32 μM vs 20 μM respectively). Despite their structural similarity 4μ8C caused a large increase in XBP1u expression when compared with the untreated and MKC-8866 (and proposed to be much more specific) treated MCF7 cells. This is not the first time that 4μ8C has been proposed to have off-target effects with the potential to activate other pathways and demonstrates how multiplexed assessment can allow for mechanistic insights [40, 41]. In this case, simultaneous detection of XBP1u and XBP1s demonstrated that in MCF7 models 4μ8C not only reduces XBP1 splicing but also increases stability or expression of full length XBP1u.

Considering the reported antagonistic effects of XBP1u and XBP1s their simultaneous measurement could provide an increased understanding of their protein-protein relationship and how these interactions govern the downstream effects observed [9]. Previous studies focusing only on XBP1s expression may have failed to take XBP1u’s known p53-p21, temporary translational arrest and inhibitory effects (beyond degradation) on XBP1s transcriptional activity into account [8–10, 42]. Here we have demonstrated how XBP1u protein varies greatly dependent on the model system used (and perhaps tissue assessed) while the implications of such protein level expression levels are yet to be explored.

Quantitative detection of basal and treatment–induced/inhibited XBP1 splicing, at the protein level in a high throughput amenable format presents an opportunity for pre-clinical and clinical applicability of XBP1 isoforms as an accessible biomarker. The XBP1 biochip also appears to show significant advantages over immunoblotting, not only in turn-around time, but in applicability to non-adherent model systems. Detection of protein level changes in circulating cells due to *XBP1* splicing and its inhibition could have great benefits in upcoming clinical trials, where non-invasive monitoring of IRE1α or XBP1 targeting therapies efficacy will be required.

In comparison to other available methods, the XBP1 biochip allows for much greater in-depth analysis, providing simultaneous quantitative measurement of both isoforms. To date no other commercially available method can simultaneously quantify the two specific XBP1 protein isoforms while the XBP1 biochip is able to do this from an individual sample in approximately 3 h. This multiplexed quantitative analysis provides a tool for all researchers studying both or either of the XBP1 isoforms in a large variety of disease contexts, affording opportunities to monitor drug efficacy, confirm protein level changes in expression in relevant models and stratify patients for targeted therapies to name but a few applications.

## Conclusion

In conclusion, we have shown here the development and utility of a rapid, sensitive, quantitative and multiplexed immunoassay array for detection of the two XBP1 isoforms. With a turn-around time of ~ 3 h, and simultaneous, quantitative analysis, it provides efficient and objective results which are not currently possible through any other commercially available method. We anticipate that this novel method of XBP1 isoform detection will make routine clinical screening feasible for multiple future diagnostic applications.

## Supplementary information

**Additional file 1: Figure S1.** *A sandwich immunoassay which captured the conventionally (conv.) spliced or frameshifted C-terminus of the XBP1 isoforms and a pan-detector of the common N-terminus was designed*. Due to the unconventional splicing of *XBP1* mRNA a translational frameshift occurs and results in XBP1 isoforms of differing length and C-terminal sequences (A). A biochip was proposed that utilised these different C-termini for simultaneous capture and the common N-terminus for detection (B).

**Additional file 2: Table S1.** *Each DTR produces an antigen dependent signal not related to levels of the other analyte.* 2.5 ng/ml of XBP1s and XBP1u were individually spiked into calibrator base and serially diluted. Acceptable background is ≤100 RLUs in the non-target DTR.

**Additional file 3: Figure S2.** *XBP1 protein ratio upon pharmacologically induced ER stress.* XBP1 levels in MDA-MB-231 cells, expressed as a ratio of XBP1s (pg/mg)/XBP1u (pg/mg), upon induction with Tg (0.5 mM, 48 h), Tm (1 μg/ml, 16 h), BFA (0.5 μg/ml, 16 h) and DTT (4 mM, 2 h).

**Additional file 4: Figure S3.** *Pre-clinical models of TNBC show results corelating with individual analyte assessment when assessed by XBP1 ratio.* XBP1s and XBP1u levels expressed as a ratio of XBP1s (pg/mg)/XBP1u (pg/mg) in MCF7, SKBR3 and MDA-MB-231 cell lines (A). XBP1s and XBP1u levels expressed as a ratio of XBP1s (pg/mg)/XBP1u (pg/mg) in MCF7 and MDA-MB-231 cells treated with vehicle (DMSO) or IRE1α RNase inhibitors 4μ8C (32 μM) or MKC-8866 (20 μM) for 48 h and assessed XBP1 biochip (B). XBP1s and XBP1u levels expressed as a ratio of XBP1s (pg/mg)/XBP1u (pg/mg) in MDA-MB-231 cells following 48 h treatment with XBP1 splicing inducing chemotherapeutic Paclitaxel (10 nM) and IRE1α RNase inhibition (C).

**Additional file 5: Figure S4.** *Loading of Lysate and diluted recombinant protein in* Fig. 4 *was confirmed by silver staining***.** Recombinant XBP1 isoforms were diluted in ddH_2_O before addition of 5X Laemmli buffer. A serial dilution was run alongside unstimulated and 0.5 μM Tg stimulated U937 lysate and silver stained (A). RIPA lysed U937 cells were treated as in (A) and assessed by XBP1 biochip. XBP1s and XBP1u levels expressed as a ratio of XBP1s (pg/mg)/XBP1u (pg/mg). ** *p < 0.01* (B).

**Additional file 6: Figure S5.** *Assessment of XBP1s/u protein ratio is not indicative of IRE1α activity in THP-1 cells*. XBP1s and XBP1u levels in THP-1 cells (stimulated to induce NLRP3 inflammasome activation and inhibited by MKC-8866) expressed as a ratio of XBP1s (pg/mg)/XBP1u (pg/mg). ** *p < 0.01*.

## Data Availability

The datasets measured and analyzed during the study are available from the corresponding authors upon reasonable request.

## Abbreviations

ATF6: Activating Transcription Factor 6
BAT: Biochip Array Technology
BCA: Bicinchoninic acid
DTR: Discrete Test Region
ELISA: Enzyme-linked immunosorbent assay
ER: Endoplasmic Reticulum
HRP: Horse Radish Peroxidase
IRE1: Inositol Requiring Enzyme 1
NLRP3: NACHT, LRR and PYD domains
PERK: Protein kinase RNA-activated (PKR)-like ER Kinase
RLU: Relative Light Unit
TAE: Tris Base, Acetic Acid, EDTA
TNBC: Triple Negative Breast Cancer
UPR: Unfolded Protein Response
XBP1: X-Box binding protein 1
