# Supplementary figures and images for: Application of a New Multiplexed Array for Rapid, Sensitive, Simultaneous and Quantitative Assessment of Spliced and Unspliced XBP1
Source: Biol Proced Online. 2019 Nov 15;21:22. doi: 10.1186/s12575-019-0111-3 (PMC6857227; doi:10.1186/s12575-019-0111-3)

**A**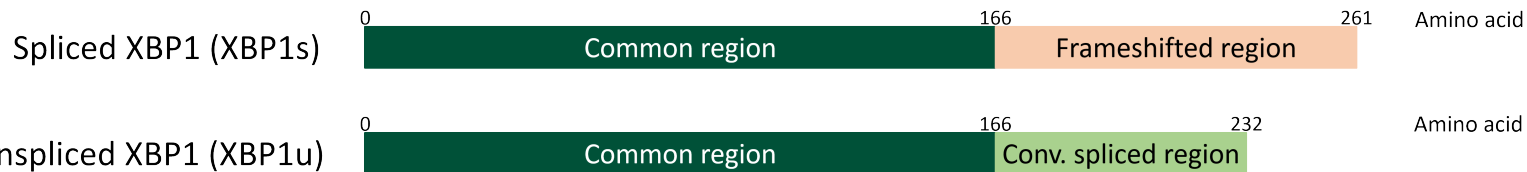**B**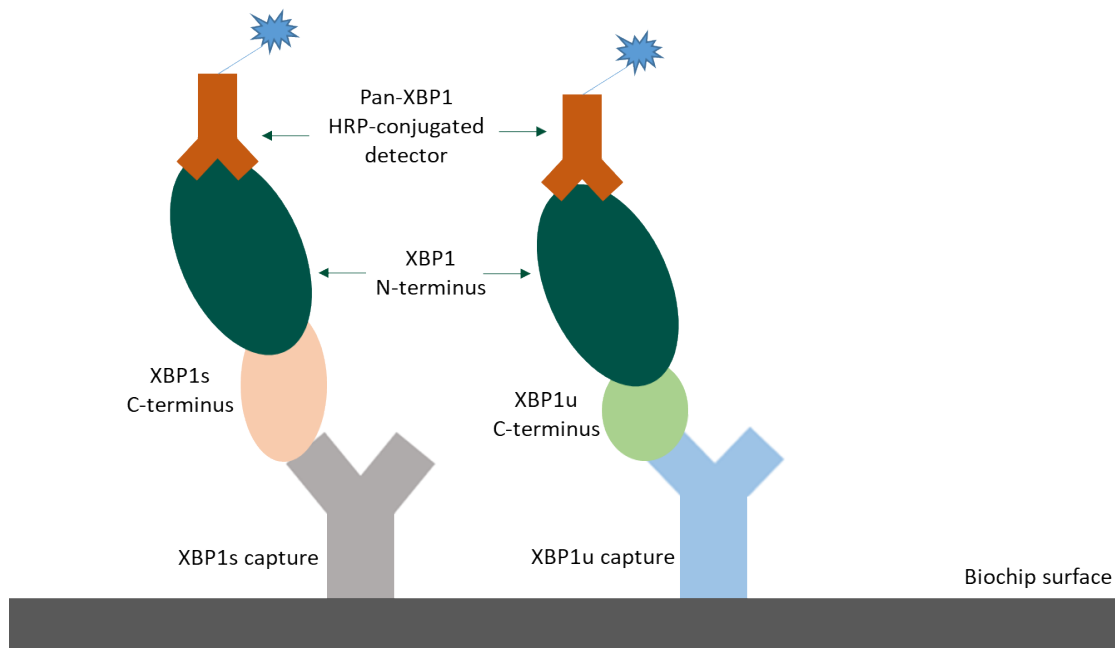

Supplement: Supplementary file 1 — Additional file 1: Figure S1. A sandwich immunoassay which captured the conventionally (conv.) spliced or frameshifted C-terminus of the XBP1 isoforms and a pan-detector of the common N-terminus was designed. Due to the unconventional splicing of XBP1 mRNA a translational frameshift occurs and results in XBP1 isoforms of differing length and C-terminal sequences (A). A biochip was proposed that utilised these different C-termini for simultaneous capture and the common N-terminus for detection (B). [file 12575_2019_111_MOESM1_ESM.pdf]

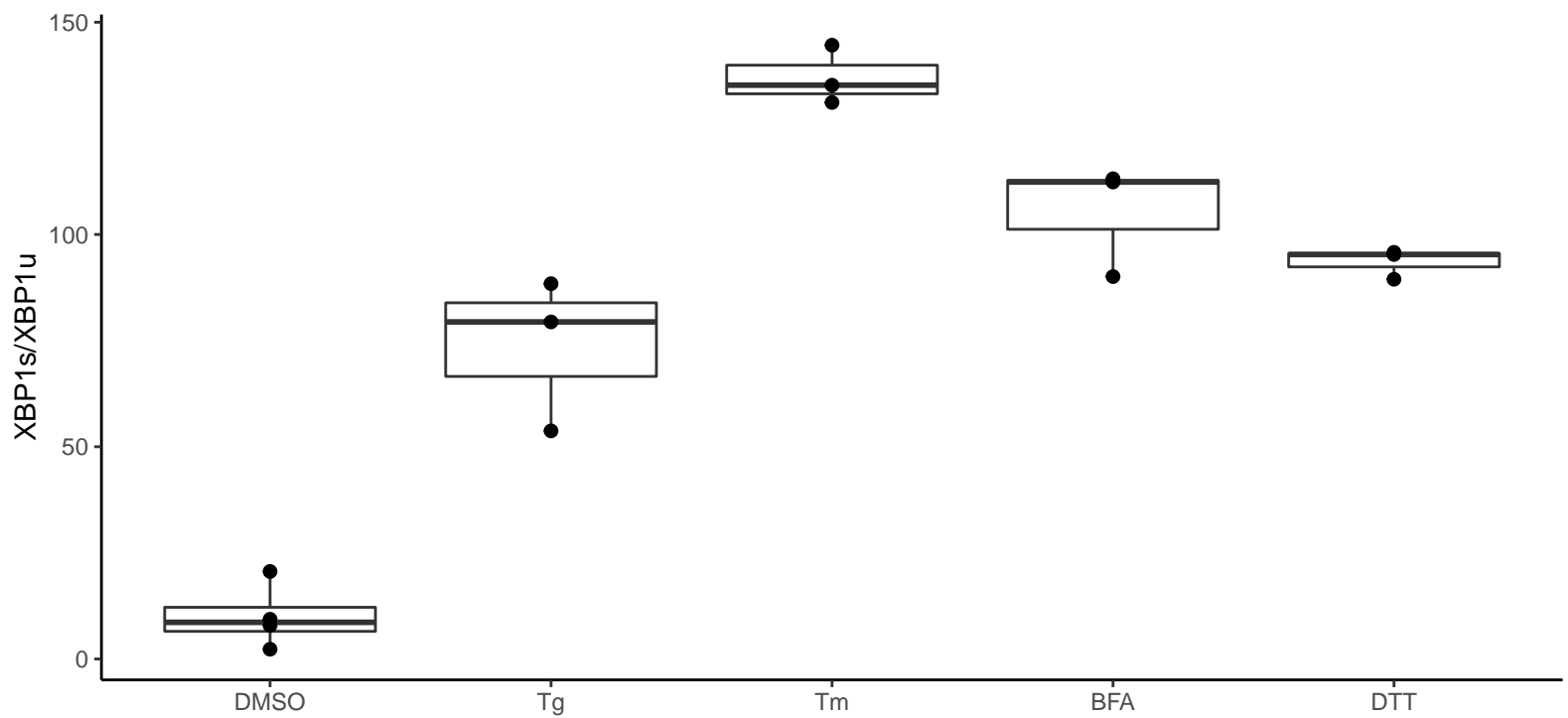

Supplement: Supplementary file 3 — Additional file 3: Figure S2. XBP1 protein ratio upon pharmacologically induced ER stress. XBP1 levels in MDA-MB-231 cells, expressed as a ratio of XBP1s (pg/mg)/XBP1u (pg/mg), upon induction with Tg (0.5 mM, 48 h), Tm (1 μg/ml, 16 h), BFA (0.5 μg/ml, 16 h) and DTT (4 mM, 2 h). [file 12575_2019_111_MOESM3_ESM.pdf]

**A**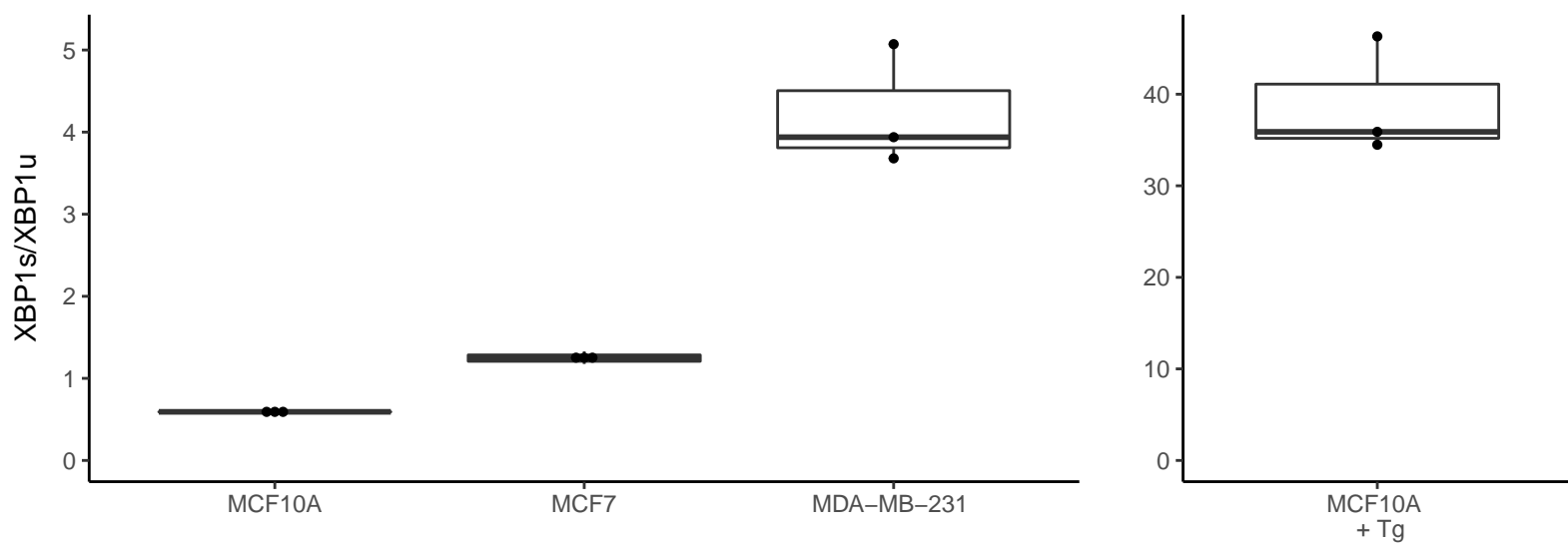**B**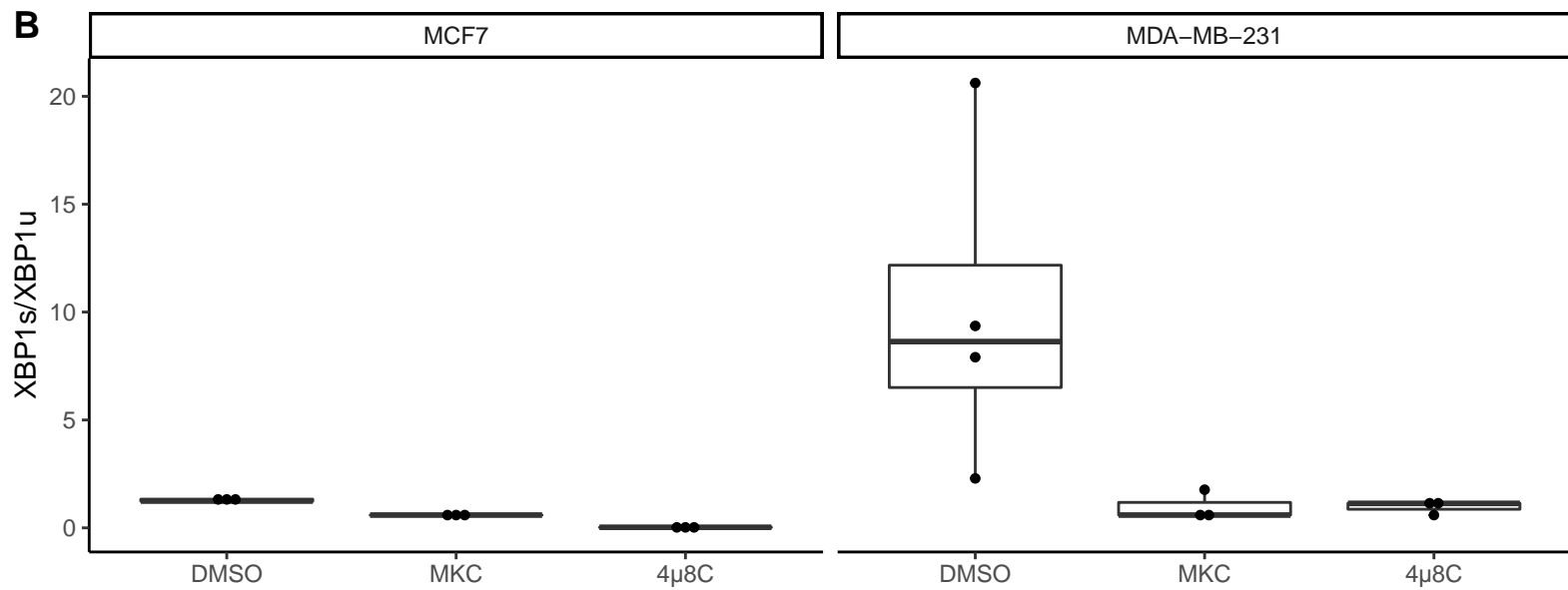**C**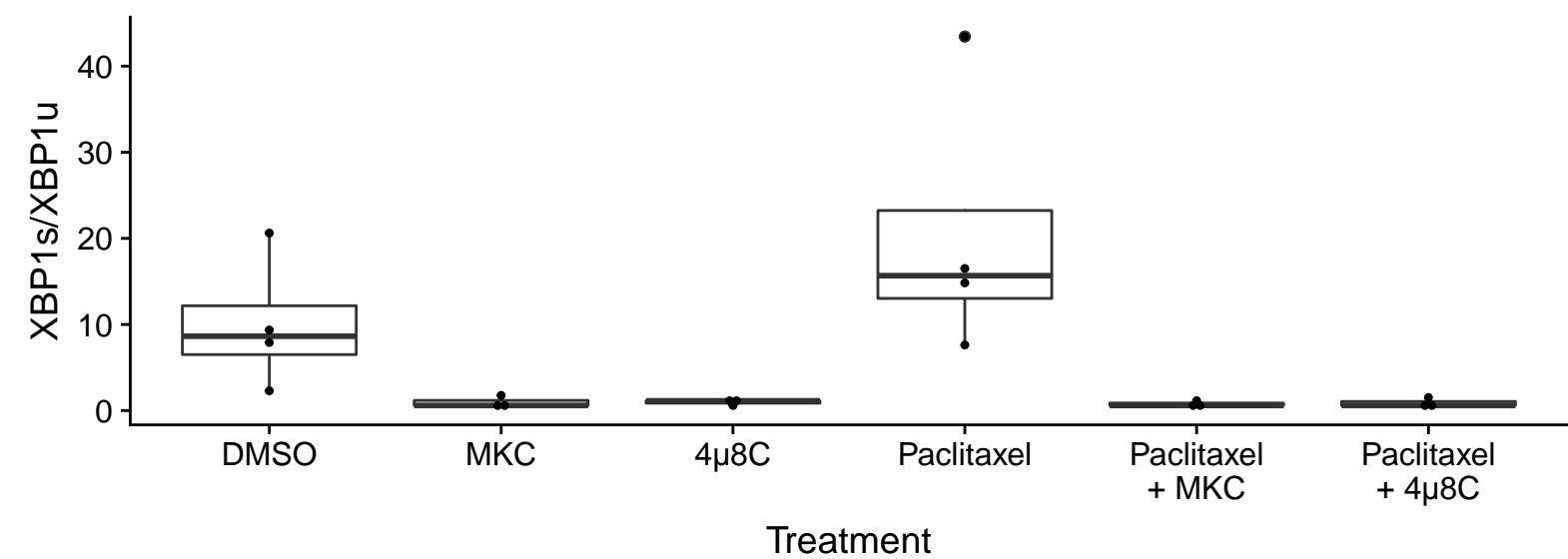

Supplement: Supplementary file 4 — Additional file 4: Figure S3. Pre-clinical models of TNBC show results corelating with individual analyte assessment when assessed by XBP1 ratio. XBP1s and XBP1u levels expressed as a ratio of XBP1s (pg/mg)/XBP1u (pg/mg) in MCF7, SKBR3 and MDA-MB-231 cell lines (A). XBP1s and XBP1u levels expressed as a ratio of XBP1s (pg/mg)/XBP1u (pg/mg) in MCF7 and MDA-MB-231 cells treated with vehicle (DMSO) or IRE1α RNase inhibitors 4μ8C (32 μM) or MKC-8866 (20 μM) for 48 h and assessed XBP1 biochip (B). XBP1s and XBP1u levels expressed as a ratio of XBP1s (pg/mg)/XBP1u (pg/mg) in MDA-MB-231 cells following 48 h treatment with XBP1 splicing inducing chemotherapeutic Paclitaxel (10 nM) and IRE1α RNase inhibition (C). [file 12575_2019_111_MOESM4_ESM.pdf]

**A**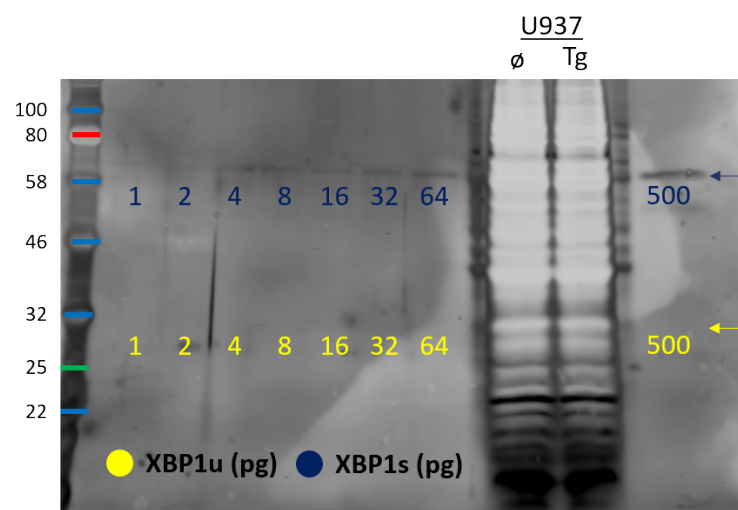**B**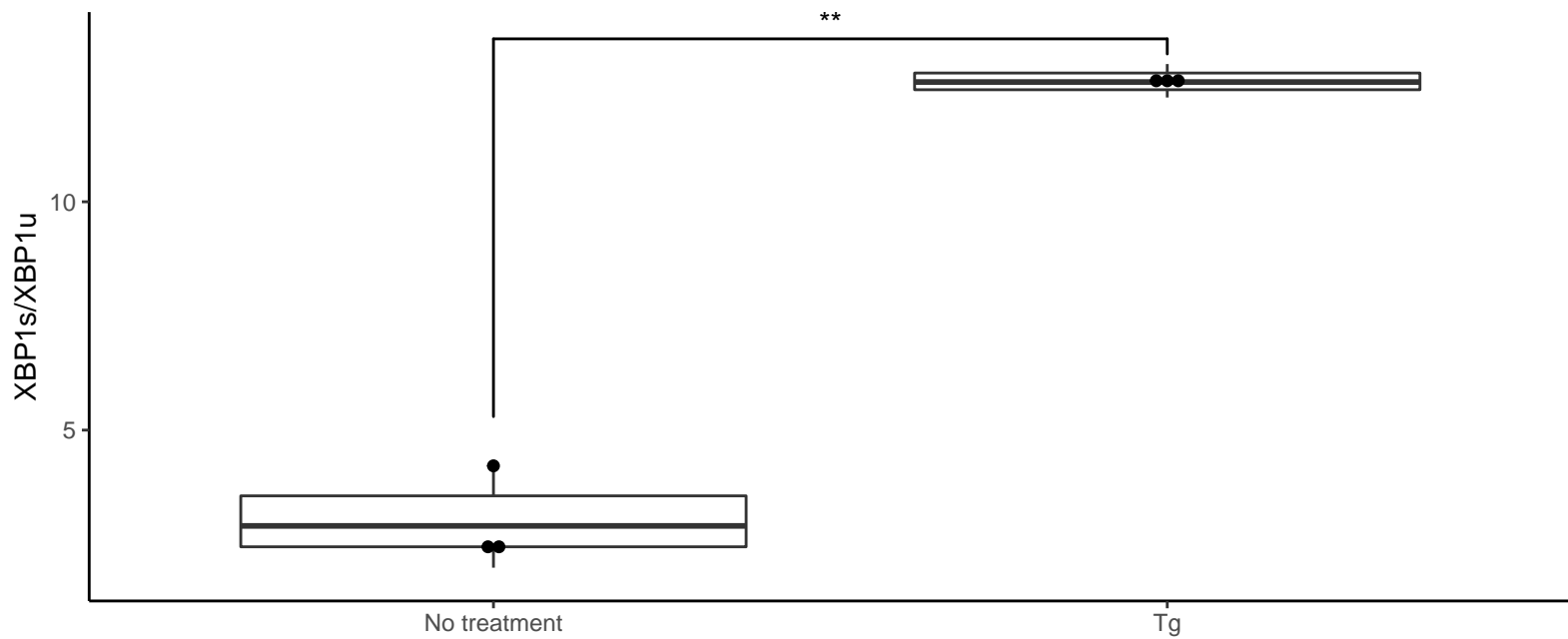

Supplement: Supplementary file 5 — Additional file 5: Figure S4. Loading of Lysate and diluted recombinant protein in Fig. 4 was confirmed by silver staining. Recombinant XBP1 isoforms were diluted in ddH2O before addition of 5X Laemmli buffer. A serial dilution was run alongside unstimulated and 0.5 μM Tg stimulated U937 lysate and silver stained (A). RIPA lysed U937 cells were treated as in (A) and assessed by XBP1 biochip. XBP1s and XBP1u levels expressed as a ratio of XBP1s (pg/mg)/XBP1u (pg/mg). ** p < 0.01 (B). [file 12575_2019_111_MOESM5_ESM.pdf]

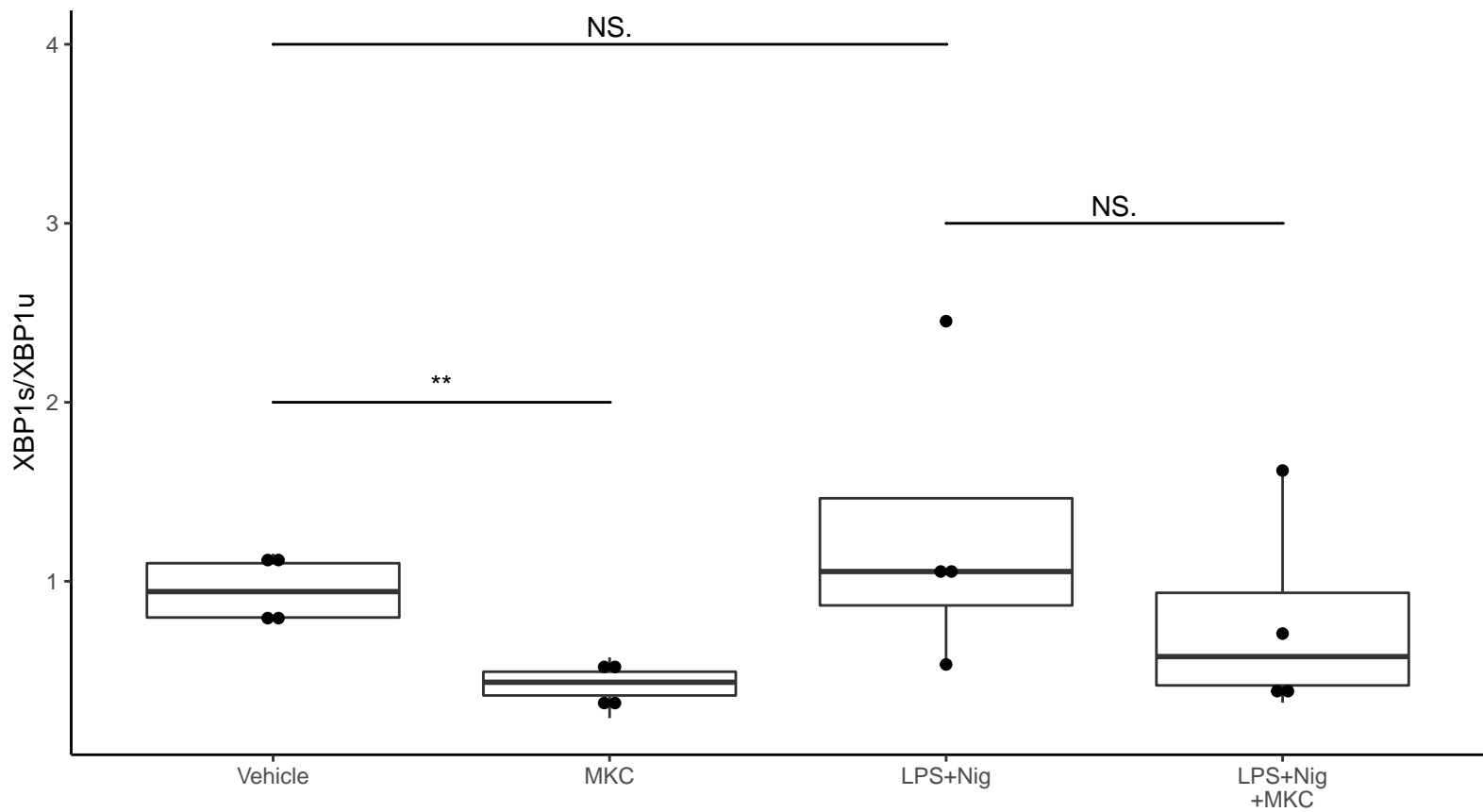

Supplement: Supplementary file 6 — Additional file 6: Figure S5. Assessment of XBP1s/u protein ratio is not indicative of IRE1α activity in THP-1 cells. XBP1s and XBP1u levels in THP-1 cells (stimulated to induce NLRP3 inflammasome activation and inhibited by MKC-8866) expressed as a ratio of XBP1s (pg/mg)/XBP1u (pg/mg). ** p < 0.01. [file 12575_2019_111_MOESM6_ESM.pdf]
