# Supplementary material for: Application of a New Multiplexed Array for Rapid, Sensitive, Simultaneous and Quantitative Assessment of Spliced and Unspliced XBP1
Source: Biol Proced Online. 2019 Nov 15;21:22. doi: 10.1186/s12575-019-0111-3 (PMC6857227; doi:10.1186/s12575-019-0111-3)
Supplement: Supplementary file 2 — Additional file 2: Table S1. Each DTR produces an antigen dependent signal not related to levels of the other analyte. 2.5 ng/ml of XBP1s and XBP1u were individually spiked into calibrator base and serially diluted. Acceptable background is ≤100 RLUs in the non-target DTR. [file 12575_2019_111_MOESM2_ESM.pdf]

A

[XBP1u] (ng)	DTR 8 (XBP1s)	DTR 14 (XBP1u)
0.000	**38**	59
0.020	**44**	286
0.039	**64**	485
0.078	**45**	958
0.156	**54**	1944
0.312	**52**	4033
0.625	**39**	8602
1.250	**64**	16254
2.500	**91**	33136

B

[XBP1s] (ng)	DTR 8 (XBP1s)	DTR 14 (XBP1u)
0.000	64	**97**
0.020	620	**68**
0.039	1200	**86**
0.078	2466	**66**
0.156	4876	**85**
0.312	10779	**73**
0.625	27219	**100**
1.250	48714	**81**
2.500	100067	**92**
